# Aggressive intracranial meningioma associated with multiple sclerosis: A case report and literature review

**DOI:** 10.3892/mi.2025.243

**Published:** 2025-05-21

**Authors:** Tirath Patel, Iram Fatima, Sai Harini Chandrasekaran, Sai Sakthi Rameshkumar, Hend Makky, Sofia Ali, Soumya Suvra Patra, Yusra Qamar

**Affiliations:** 1 Trinity Medical Sciences University School of Medicine, Kingstown VC0100, Saint Vincent and the Grenadines; 2 Holy Name Medical Center, Teaneck, NJ 07666, USA; 3 Government Medical College Omandurar, Chennai, Tamil Nadu 600002, India; 4 Faculty of Medicine, Assiut University, Assiut 71515, Egypt; 5 Peninsula Medical School, University of Plymouth, Derriford Road, Plymouth PL6 8BT, UK; 6 Calcutta National Medical College, Kolkata, West Bengal 700014, India; 7 Department of Obstetrics and Gynecology, King George's Medical University, Lucknow, Uttar Pradesh 226003, India

**Keywords:** meningioma, multiple sclerosis, radiotherapy, demyelination, magnetic resonance imaging

## Abstract

Meningiomas are the most common primary tumors of the central nervous system (CNS) and often require radiation therapy (RT), which can lead to the development of complications, such as radiation-induced demyelination. The present study describes the case of a 40-year-old male patient who developed demyelinating lesions following RT for a WHO grade I meningioma, ultimately diagnosed with MS. This condition shares clinical and radiological features with multiple sclerosis, including damage to oligodendrocytes, which are the cells responsible for myelination in the CNS, cognitive impairment, motor dysfunction, and white matter abnormalities on magnetic resonance imaging (MRI). Distinguishing between radiation-induced demyelination and multiple sclerosis can be challenging, particularly in patients with a history of radiation therapy, as MRI findings often overlap. Additionally, radiation therapy may trigger or exacerbate multiple sclerosis in individuals with pre-existing risk factors or undiagnosed multiple sclerosis. A thorough clinical history, advanced neuroimaging, and careful differential diagnosis are essential for accurate management of these conditions.

## Introduction

Meningiomas are the most common tumors of the primary central nervous system (CNS), accounting for ~37.6% of such tumors and 50% of all benign brain tumors. They arise from meningothelial (arachnoid) cells and are classified into three WHO grades based on histopathological features. Grade I meningiomas, constituting >80% of cases, are benign, while grades II and III cases exhibit increased mitotic activity, necrosis and a greater metastatic potential (1,2). These slow-growing tumors can develop from any intracranial or spinal dural surface, with symptoms varying by location, commonly presenting as headaches, seizures, or focal neurological deficits (3).

Treatment options include observation, surgical resection and radiotherapy (RT), with chemotherapy reserved for rare cases. RT is primarily used for inoperable or recurrent tumors, with doses of ~50 Gy for grade I and ~60 Gy for grade II/III tumors delivered via external beam radiotherapy or stereotactic radiosurgery (3,4). While effective, the use of RT is associated with risks, including radiation-induced brain edema, cognitive decline, necrosis and demyelination (5).

Radiation-induced demyelination results from axonal degeneration, vascular damage and blood-brain barrier disruption, mimicking multiple sclerosis (MS) in clinical and imaging findings (6). MS is an immune-mediated inflammatory disorder causing CNS demyelination and axonal injury, diagnosed using the McDonald criteria, which emphasize lesion dissemination in time and space, supported by magnetic resonance imaging (MRI), cerebrospinal fluid (CSF) analysis and oligoclonal band detection (7,8). Distinguishing MS from radiation-induced demyelination is challenging, as both conditions exhibit white matter abnormalities on MRI. Advanced neuroimaging and CSF analysis are crucial for accurate diagnosis to avoid delayed MS treatment or unnecessary interventions (9,10).

The present study describes the case of a 40-year-old male patient who developed demyelinating lesions following RT for a WHO grade I meningioma, ultimately diagnosed with MS. This case raises critical questions as regards whether radiation serves as a potential trigger for MS in predisposed individuals or if the observed changes reflect radiation-induced demyelination. Through a detailed clinical, radiological and pathological assessment, the case described herein highlights the challenges in distinguishing these conditions and emphasizes the importance of an accurate diagnosis in guiding treatment decisions.

## Case report

**Figure 1:**
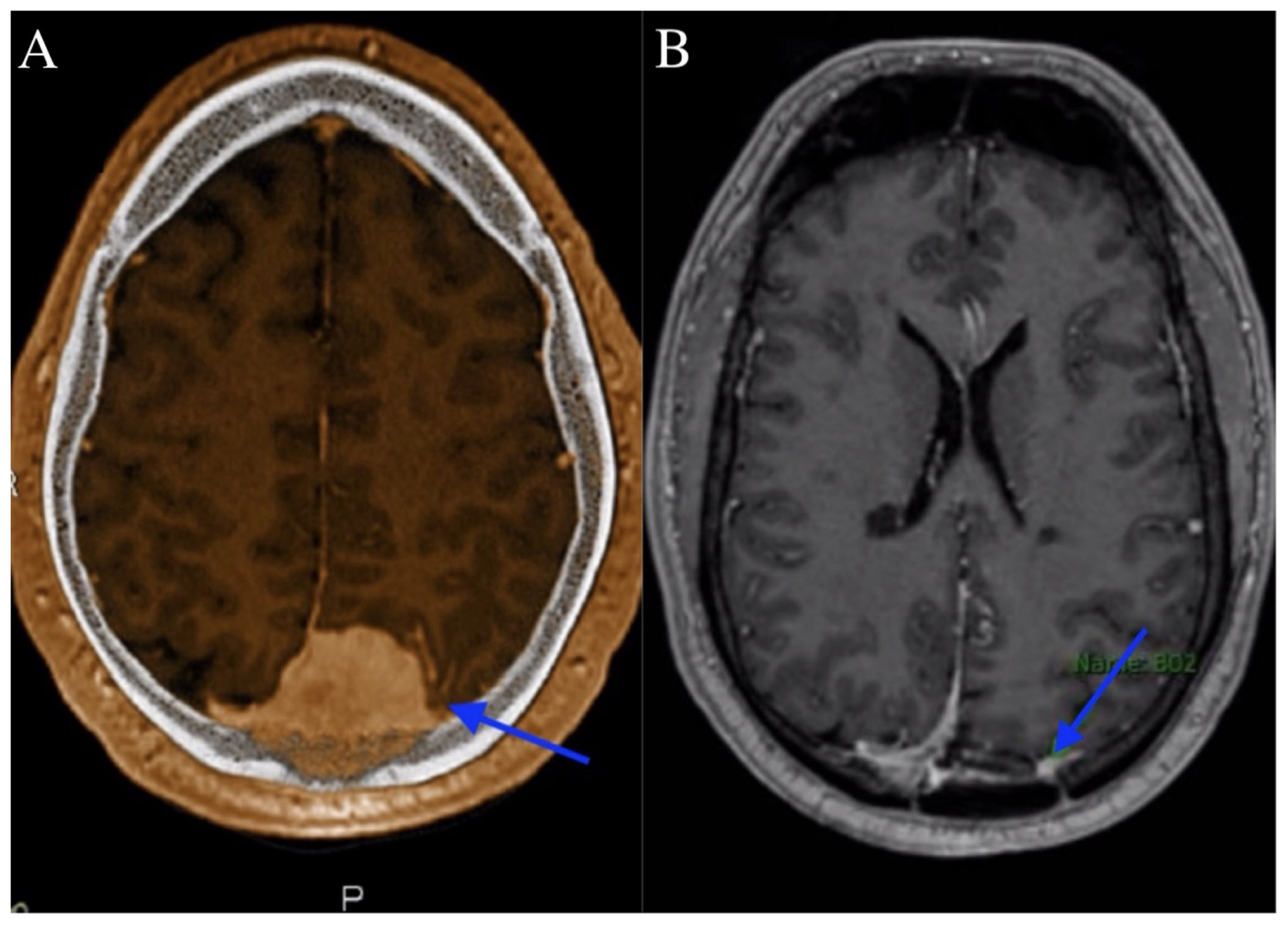
(A) Magnetic resonance imaging post-contrast illustrates an increased-size meningioma with dural thickening, left parieto-occipital vasogenic edema, and stable white matter hyperintensities (blue arrow), (B) while post-surgical imaging reveals residual meningioma with calvarial and sagittal sinus invasion, a stable resection cavity, and a small extra-axial fluid collection (blue arrow).

A 40-year-old male with a history of left occipital WHO grade I meningioma, characterized by extensive bone invasion and an elevated Ki-67 index, presented to Government Medical College Omandurar Hospital, Chennai, India. His clinical course was complicated by the subsequent diagnosis of MS. The patient initially presented with worsening chronic headaches and associated visual deficits over a period of several weeks, prompting an MRI of the brain, which revealed a 4.5-cm left occipital dural-based lesion concerning for meningioma, as shown in Fig. 1A. A pre-operative MRI scan revealed the progressive enlargement of the lesion along with increasing vasogenic edema over the following months. The patient underwent a left occipital craniectomy to debulk the parafalcine tumor.

A pathological examination confirmed a WHO grade I meningioma with significant bone invasion, a high Ki-67 index and molecular characteristics indicative of a more aggressive subtype. Immunohistochemical analysis, performed at the Health Plus Laboratory, Chennai, India, revealed strong positivity for epithelial membrane antigen and somatostatin receptor 2A, further supporting the diagnosis. The patient is under the care of the Department of Neurology at Government Medical College Omandurar Hospital, Chennai, India. It should be noted that the immunohistochemical staining was performed externally at Health Plus Laboratory, Chennai, which is not affiliated with the authors' institutions. The findings were reported by the laboratory and integrated into the patient's diagnostic workup. The authors did not perform the staining themselves. Representative images are unavailable, as the laboratory did not provide digital slides or photomicrographs. However, the results were reviewed and corroborated with clinical and radiological findings by the treating team.

**Figure 2:**
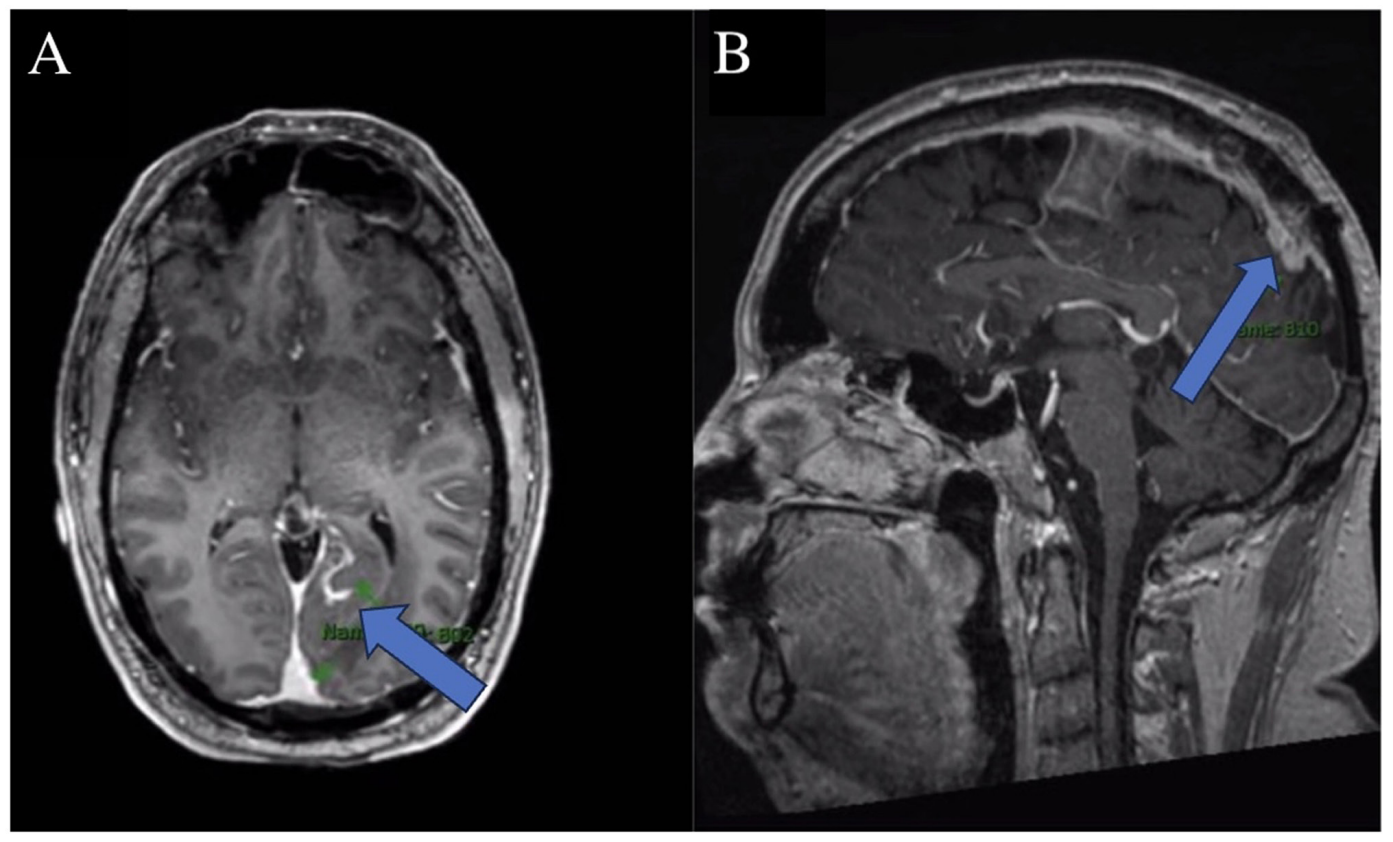
Magnetic resonance imaging illustrating residual/recurrent biparietal meningioma with calvarial and sagittal sinus invasion (blue arrows), stable demyelinating disease, and no new lesions on both (A) axial and (B) sagittal sections.

Post-surgical imaging revealed residual/recurrent biparietal meningioma with calvarial and sagittal sinus invasion, along with a stable resection cavity and a small extra-axial fluid collection, as shown in Fig. 1B. No new lesions or mass effect were noted. Post-surgery, the patient completed radiation treatment delivering a total of 54 Gy in 30 fractions, followed by a 6 Gy boost in 3 fractions, in line with the treatment protocol for WHO grade I meningioma. Following treatment, he experienced worsening left facial numbness, ataxia and the emergence of new enhancing lesions, leading to a diagnosis of MS, as shown in Fig. 2.

Further diagnostic evaluation for MS included CSF analysis, which revealed the presence of IgG oligoclonal bands and an elevated IgG index, CNS inflammation. These findings indicated intrathecal IgG synthesis, consistent with the inflammatory and demyelinating processes characteristic of MS. Visual evoked potentials (VEPs) demonstrated delayed conduction, further reinforcing the diagnosis of MS. The detailed CSF analysis results are summarized in Table I.

The presence of four distinct IgG oligoclonal bands in the CSF, absent in the serum, strongly supported the diagnosis of MS, reflecting immune activity within the central nervous system. The elevated IgG index (0.92) indicated increased IgG production relative to albumin, a finding observed in patients with MS. Mildly elevated CSF total protein levels and a slight increase in white blood cells (pleocytosis) are also consistent with the inflammatory profile of MS, although these are less specific. These clinicopathological data, combined with clinical symptoms, the VEP findings and MRI evidence of demyelinating lesions, align with the McDonald criteria for the diagnosis of MS.

The most recent MRI, performed at 3 months following the completion of RT, revealed a stable appearance of demyelinating disease, with no new lesions or abnormal enhancement indicative of active demyelination. At his last follow-up with neurosurgery and neurology, the patient denied any prolonged episodes of nausea/vomiting, headaches, seizures, diplopia, changes in hearing, speech disturbances, gait disturbances or falls, focal motor/sensory deficits, or dysphagia.

## Discussion

RT can lead to a range of harmful effects on nervous tissue, which can be classified into three categories based on the timing of onset. Acute complications develop within days to weeks, early delayed complications arise between 1 to 6 months, and late-delayed complications emerge after >6 months (11).

Demyelination is a known complication of RT which belongs to the early-delayed category. The possible pathogenesis of radiation-induced demyelination is due to the direct effects of radiation on oligodendrocytes, as well as oligodendrocyte-type-2-astrocyte progenitor cells, which serve as progenitor cells for oligodendrocytes and type-2 astrocytes. Radiation also affects vasculature and blood brain barrier and alters cytokine expression, which together, may play a role in pathogenesis (12). MS is an inflammatory immune disorder. Similar to radiation-induced demyelination, in MS, oligodendrocytes are considered to be the primary target through which various mechanisms lead to the loss of myelin and impaired nerve conduction (13).

The diagnosis of MS is primarily based on the McDonald criteria, which emphasize the demonstration of dissemination of lesions in both time and space, supported by clinical symptoms, MRI findings and CSF analysis, particularly the presence of oligoclonal bands (14). By contrast, radiation-induced demyelination is characterized by localized white matter changes in irradiated regions, often without evidence of dissemination. In the patient in the present study, the presence of multiple new enhancing lesions, independent of the irradiated field, alongside clinical progression, was more suggestive of MS rather than radiation-induced demyelination. However, additional diagnostic markers, including CSF analysis, could have further strengthened this distinction.

Several case reports and studies have described radiation-induced demyelination as a delayed complication of cranial irradiation, typically presenting months to years post-treatment (15). Pathophysiologically, radiation damages oligodendrocytes and disrupts the blood-brain barrier, leading to a cascade of inflammatory responses that can mimic MS. While some studies have suggested that radiation may unmask or accelerate MS in predisposed individuals, direct causation remains unproven (16). The clinical history of the patient, a lack of prior demyelinating episodes and the pattern of lesion development necessitate a cautious approach in determining whether radiation plays a contributory role in the onset of MS. The patient described herein was diagnosed with MS after he received RT for meningioma.

Recent advancements in imaging and biomarker research have improved the ability to of medical professionals to differentiate MS from radiation-induced changes. Studies such as those reported in Visualizing Emerging Worlds (VIEW) 2024 and ACS Nano (American Chemical Society Nano) have explored novel imaging modalities and molecular markers that could aid in distinguishing these conditions (17). Advanced MRI techniques, including diffusion tensor imaging (DTI) and susceptibility-weighted imaging (SWI), provide additional insights into microstructural changes unique to radiation-induced damage vs. MS-related demyelination. DTI assesses white matter integrity by measuring water diffusion along axonal tracts, which helps differentiate MS plaques from radiation-induced lesions, while SWI enhances the visualization of iron deposition and microvascular abnormalities, features more commonly observed in MS due to chronic inflammation and neurodegeneration (18,19). The future integration of these imaging techniques, along with molecular biomarkers, could refine diagnostic accuracy and guide more personalized treatment strategies, ultimately improving patient outcomes.

Radiation has been shown to have increased neurotoxic effects in patients with MS or patients who are predisposed to the disease (13,20). RT alone does not lead to MS, but it can encourage the development of flares of MS. The reduced expression of ataxia-telangiectasia serine/threonine kinase is observed in some patients with MS. This gene is involved in DNA repair pathways; thus, its decreased expression can enhance the sensitivity to radiation (21). Thus, it may be possible that the patient in the present study could have had undiagnosed MS prior to receiving RT, or perhaps he possessed the risk factors that predisposed him to developing MS. The dose-effect association between radiation and demyelination in patients with MS was evaluated in a previous study. It was shown that all MS lesions occurred in brain regions irradiated with an intermediate dose; i.e., a mean biologically effective dose (BED_2_) of 33.9 Gy (27.3-49.6 Gy) (10). Caution should be taken when considering RT for patients with MS or who possess risk factors for the disease. MS can appear as radionecrosis and metastases on imaging; thus, differential diagnoses should be kept in mind to avoid mistakes in diagnosis and initiating the correct treatment promptly.

Radiation-induced demyelination can mimic or trigger MS, posing diagnostic and management challenges. Prevention focuses on minimizing radiation exposure to healthy brain tissue using advanced techniques, such as intensity-modulated radiotherapy and proton therapy (22). Treatment varies based on severity; corticosteroids help reduce acute inflammation, while disease-modifying therapies are considered in confirmed cases of MS. Emerging neuroprotective and remyelination-promoting agents show promise, and severe cases may benefit from plasmapheresis or intravenous immunoglobulin (23). Prognosis is variable, with some patients stabilizing and others experiencing progression.

The present study outlines key histopathological and immunohistochemical findings; however, it is limited by the lack of immunohistochemistry microscopy images and surgical or pathological photos, due to restricted access and unavailable records. Despite this, it is considered that the diagnosis is well-supported by detailed histological and molecular data.

In conclusion, RT for meningiomas can lead to complications, such as radiation-induced demyelination, which mimics MS in both clinical symptoms and MRI findings. Distinguishing between these conditions is challenging, paritcularly in patients with a history of radiation, as radiation may also trigger or worsen MS in predisposed individuals. Accurate diagnosis requires a thorough clinical history, advanced imaging, and careful differential diagnosis. A multidisciplinary approach is essential for effective management and optimal patient outcomes.

## Figures and Tables

**Table I:** Results of CSF analysis.

Parameter	Result	Reference range	Interpretation
IgG oligoclonal bands	Positive (4 bands)	Negative (0 bands)	Abnormal, suggestive of MS
IgG index	0.92	0.34-0.66	Elevated, supports the diagnosis of MS
CSF total protein	48 mg/dl	15-45 mg/dl	Mildly elevated
CSF glucose	60 mg/dl	40-70 mg/dl	Normal
White blood cell count	8 cells/µl	0-5 cells/µl	Mild pleocytosis

CSF, cerebrospinal fluid.

## Data Availability

The data generated in the present study may be requested from the corresponding author.
